# Genome Sequence of *Mycoplasma parvum* (Formerly *Eperythrozoon parvum*), a Diminutive Hemoplasma of the Pig

**DOI:** 10.1128/genomeA.00986-13

**Published:** 2013-11-27

**Authors:** Naíla C. do Nascimento, Andrea P. dos Santos, Yuefeng Chu, Ana M. S. Guimaraes, Apryl Pagliaro, Joanne B. Messick

**Affiliations:** Department of Comparative Pathobiology, Purdue University, West Lafayette, Indiana, USAa; State Key Laboratory of Veterinary Etiological Biology, Lanzhou Veterinary Research Institute of CAAS, Lanzhou, Chinab

## Abstract

We report the complete genome sequence of *Mycoplasma parvum* strain Indiana. Its circular chromosome is 564,395 bp, which is smaller than that of *Mycoplasma genitalium*, which was previously considered the smallest member of the *Mollicutes*. Comparative analyses of the genomes of *M. parvum* and *Mycoplasma suis* will provide novel insights into the molecular basis of their virulence.

## GENOME ANNOUNCEMENT

*Mycoplasma parvum* (formerly *Eperythrozoon parvum*) is the only remaining species of *Eperythrozoon* whose name has standing in nomenclature and has not yet been examined by molecular genetic methods (1). This nonpathogenic bacterium of pigs, described by Splitter in 1950, is reported to often accumulate in large numbers on a single red blood cell (RBC); however, only a few RBCs are infected. It is seldom >0.5 µm in size. In contrast, *Mycoplasma suis*, the other hemoplasma known to infect pigs, varies in size from 1 µm to 2.5 µm, and its massive bacteremia, characterized by fever and icteroanemia, may result in death. The presence of two separate species in pigs was established on the basis of differences in morphology and pathogenicity, as well as by cross-inoculation studies. Similar species of red cell bacteria that infect cattle, sheep, goats, and mice were also differentiated from the two species infecting pigs by cross-inoculation studies (2).

Genomic DNA from *M. parvum* strain Indiana was purified from the blood of a splenectomized pig, which was naturally infected with the hemoplasma, using Quick-gDNA MidiPrep (Zymo Research Corporation, Irvine, CA) according to the manufacturer’s recommendations. The whole genome was sequenced using Illumina HiSeq 2000 (Illumina, Inc., San Diego, CA) paired-end sequencing at the Purdue University Genomics Core Facility. Average reads of about 100 bases were assembled using ABySS 1.2.7. After assembly resulting from 1,000× genome coverage of the Illumina reads, the single remaining gap was closed using conventional PCR, followed by Sanger sequencing in both directions. First-pass annotation was achieved using the NCBI annotation pipeline.

The complete genome of *M. parvum* strain Indiana is composed of 564,395 bp in a single circular chromosome, with a G+C content of 27%. It appears to use the opal stop codon (UGA) for tryptophan. The general genomic features of *M. parvum* are similar to those of the hemoplasmas sequenced to date (3–10), in that the 16S, 23S, and 5S rRNA genes are represented as single copies; however, like that of *M. suis*, the 16S rRNA gene is separated from the 5S-23S rRNA in a different operon (3). Thirty-one tRNAs were identified, covering all amino acids.

A total of 581 protein-coding sequences (CDSs) were predicted, and putative functions were assigned by the NCBI annotation pipeline. One hundred thirty-one CDSs have putative functional identities; however, most (77%) of the CDSs are represented by hypothetical proteins. A large proportion (26%) of the genome is dedicated to duplicated genes organized in paralog families, mostly composed of hypothetical CDSs. The latter finding is consistent with other hemoplasmas, which have a large repertoire of paralog genes thought to participate in antigenic variation. The sequence described herein represents a valuable new resource for the study of *M. parvum* and for comparative analyses to its closest relative, *M. suis*, providing novel insights into the genetic basis of their virulence.

### Nucleotide sequence accession number.

The genome sequence of *M. parvum* strain Indiana has been deposited in GenBank under the accession no. CP006771.
